# Genome-Wide Identification, Phylogenetic Classification, and Expression Profiling of the HSF Gene Family in *Rosa hybrida* Under Heat and Drought Stress

**DOI:** 10.3390/plants14203167

**Published:** 2025-10-15

**Authors:** Jiao Zhu, Shikai Fan, Rongchong Li, Fei Dong, Yiyang Liu, Chengpeng Wang

**Affiliations:** 1 Shandong Engineering Research Center of Ecological Horticultural Plant Breeding, Institute of Leisure Agriculture, Shandong Academy of Agricultural Sciences, Jinan 250100, China; mmzhujiao520@163.com (J.Z.); dongfei860117@163.com (F.D.); 2 Shandong Provincial Key Laboratory of Crop Genetic Improvement, Ecology and Physiology, Institute of Crop Germplasm Resources, Shandong Academy of Agricultural Sciences, Jinan 250100, China; skfan09@126.com (S.F.); lirongchong@126.com (R.L.)

**Keywords:** *Rosa hybrida*, heat shock transcription factors (HSFs), heat stress, drought stress, gene expression profiling

## Abstract

*Rosa hybrida* (*R. hybrida*), a widely cultivated ornamental species, is increasingly threatened by climate-induced abiotic stresses, including heat and drought. Heat shock transcription factors (HSFs) are critical for plant stress responses, yet their roles in *R. hybrida* remain understudied. In this research, 71 HSF genes were identified from the haplotype-resolved genome of the tetraploid variety ‘Samantha’. These genes were classified into three major classes (HSFA, HSFB, HSFC) and 15 subgroups based on phylogenetic and motif analysis. Gene structure and conserved motifs revealed subgroup-specific functional divergence. Promoter analysis identified abundant hormone- and stress-responsive cis-elements, particularly for abscisic acid (ABA) and jasmonic acid. Synteny analysis suggested that segmental duplication contributed to the RhHSF family’s expansion. Tissue-specific expression profiling revealed distinct roles for HSFs, with HSFB genes predominantly expressed in reproductive tissues and HSFA genes in vegetative organs. Expression under heat and drought stress showed dynamic, subgroup-dependent responses, with HSFC members playing significant roles. Functional assays demonstrated that *RhHSF17*, induced by both stresses and ABA, localized to the nucleus, and its overexpression in Arabidopsis enhanced drought tolerance. This study provides a comprehensive characterization of the RhHSF gene family, offering insights into their roles in stress tolerance and laying the foundation for future functional research.

## 1. Introduction

Global climate change has led to increased occurrences of abiotic stresses such as drought and heat, which severely impact plant growth, development, and productivity [1]. Notably, these two stresses frequently co-occur and interact synergistically during extreme weather events, such as heatwaves [2]. The co-occurrence of heat and drought imposes compounded physiological burdens on plants, including disrupted photosynthetic capacity, oxidative stress, and metabolic imbalance [1,3,4]. These stresses can also trigger conflicting physiological pathways, exemplified by stomatal regulation, where heat promotes opening for cooling while drought enforces closure to conserve water [5,6,7]. Although such conflicts contribute significantly to plant injury, especially under prolonged stress, they also reflect the need for precise transcriptional coordination to resolve competing signals [8].

In response to environmental stress, plants have evolved complex defense systems that include the activation of transcriptional regulators such as heat shock transcription factors (HSFs). HSFs constitute a highly conserved gene family that plays a central role in modulating plant responses to various abiotic stresses, including heat and drought [9,10,11]. Typically, plant HSF proteins are characterized by several conserved functional domains, including a DNA-binding domain (DBD), an oligomerization domain (OD or HR-A/B), and a nuclear localization signal (NLS) [12]. Some HSFs also contain a nuclear export signal (NES). The DBD enables HSFs to recognize and bind specifically to heat shock elements (HSEs) in the promoter regions of target genes, thereby initiating stress-responsive transcription [13]. The HR-A/B region facilitates trimerization, which is essential for stable DNA binding and transcriptional activation [14,15]. Once activated, HSFs bind to conserved HSE motifs within gene promoters and recruit transcriptional machinery to form active transcriptional complexes [16]. Plant HSFs are typically divided into three classes—HSFA, HSFB, and HSFC—based on their distinct structural characteristics [10]. HSFA proteins primarily serve as transcriptional activators, whereas HSFB members usually function as co-regulators or transcriptional repressors [17]. In contrast, HSFC proteins remain less well studied, though emerging evidence suggests they may exert modulatory effects during stress responses [9]. These family members activate downstream stress-responsive genes, especially heat shock proteins (HSPs), which play key roles in maintaining protein stability and improving plant tolerance to environmental stress [9].

Functional studies across diverse plant species have established that HSFs act as pivotal regulators of abiotic stress responses, particularly under heat and drought conditions, which frequently co-occur in natural and agricultural environments. Among them, members of the HSFA1 subclass—such as AtHSFA1a and AtHSFA1b in *Arabidopsis thaliana* (*A. thaliana*)—function as master regulators that initiate the heat shock response. They do so by inducing the expression of molecular chaperones including HSP70, HSP101, and small heat shock proteins (sHSPs), thereby promoting protein refolding and thermotolerance [18,19,20]. Other HSFs also function in coordinating responses to thermal and ROS-mediated oxidative stress. For example, in Arabidopsis, *HsfA2* not only induces HSPs but also activates ROS-scavenging enzymes such as *APX2*, thereby preserving mitochondrial integrity under stress conditions [21]. In barley (*Hordeum vulgare*), constitutive expression of *HvHSFA2e* enhances both heat and drought tolerance by activating heat shock proteins and antioxidant enzyme genes, reducing ROS accumulation and lipid peroxidation [22]. Similarly, recent work in *Rosa chinensis* revealed that *RcHsf17* is strongly induced by heat and drought stress, underscoring its potential role in enhancing stress tolerance in roses [23]. Interestingly, recent evidence suggests that combined heat and drought stress elicits unique transcriptional programs distinct from those induced by individual stresses. In wheat, HSFA6s has been shown to mediate cross-talk between osmotic and heat signaling pathways by binding not only to heat shock elements (HSEs) but also to ABA-responsive elements (ABREs), indicating its role as a node of integration in multi-stress regulatory networks [24,25]. These findings highlight the emerging concept that certain HSFs function as stress-type integrators, fine-tuning specific downstream pathways to enhance plant survival under complex and fluctuating environmental conditions.

While HSFs have been extensively studied in model organisms such as *A. thaliana* and major crops like rice (*Oryza sativa* (*O. sativa*)) and wheat (*Triticum aestivum*), limited attention has been given to this gene family in ornamental plants. *Rosa hybrida* (*R. hybrida*), commonly known as modern rose, is one of the most economically important ornamental species globally, widely cultivated for landscaping and cut flower production [26]. However, the open-field cultivation of rose exposes it to abiotic stresses such as drought and high temperature, particularly in summer seasons and arid climates [27,28]. Despite the increasing threat of climate-induced stress, the molecular mechanisms underlying stress adaptation in rose remain limited. Recent progress in rose genomics, especially the high-quality haploid genome assembly of the tetraploid cultivar ‘Samantha’, provides a valuable genomic resource in *R. hybrida* [29]. This assembly offers new opportunities to systematically explore gene families associated with abiotic stress responses, paving the way for molecular breeding and biotechnology to improve rose stress tolerance.

In this study, we conduct a genome-wide identification and characterization of the HSF gene family in *R. hybrida*, with a focus on their potential roles in drought and heat stress responses. Comprehensive analyses, including phylogenetic classification, motif composition, gene structure, promoter cis-element profiling, and synteny evaluation, were performed to elucidate the evolutionary patterns and regulatory potential of *RhHSFs*. Furthermore, expression profiling under multiple stress treatments and functional characterization of a representative member provide novel insights into their dynamic regulatory roles. Our findings highlight the pivotal contribution of HSF family members, particularly class C regulators, in integrating hormone- and stress-mediated signaling pathways in modern rose.

## 2. Results

### 2.1. Identification of HSF Genes in Rosa hybrida

To identify members of the HSF gene family in modern rose (*R. hybrida*), we performed BLAST (ncbi-blast-v2.10.1+) searches combined with domain screening. Sequences lacking the characteristic HSF DNA-binding domain (DBD) or the HR-A/B oligomerization domain, as well as redundant or incomplete sequences, were removed. In total, 71 HSF family members were identified from the haplotype-resolved chromosomes of the tetraploid rose cultivar ‘Samantha’ (Table S1). These genes were designated *RhHSF1* to *RhHSF71*, numbered according to their physical positions along the haplotype-resolved pseudo-chromosomes (1A–7D) from top to bottom. The RhHSF proteins vary considerably in sequence length, ranging from 116 to 526 amino acids, with predicted molecular masses between 12.12 and 57.28 kDa. Theoretical isoelectric points (pI) range from 4.70 to 8.71, suggesting functional and structural diversity among family members. Chromosomal position revealed that these genes are distributed across four homologous chromosome groups, reflecting the complexity of the modern rose genome. According to in silico predictions, all RhHSF proteins are likely localized in the nucleus, consistent with their roles as transcription factors.

### 2.2. Phylogenetic Relationships and Classification of RhHSFs

To investigate the classification and phylogenetic relationships of the HSF gene family in *R*. *hybrida*, we conducted a comprehensive phylogenetic analysis of 71 RhHSF proteins, alongside representative HSF members from *A. thaliana* and *O. sativa* (Table S2). A maximum likelihood (ML) phylogenetic tree was constructed based on multiple sequence alignment of the full-length HSF protein sequences, and branch support was evaluated using 1000 bootstrap replicates.

**Figure 1:**
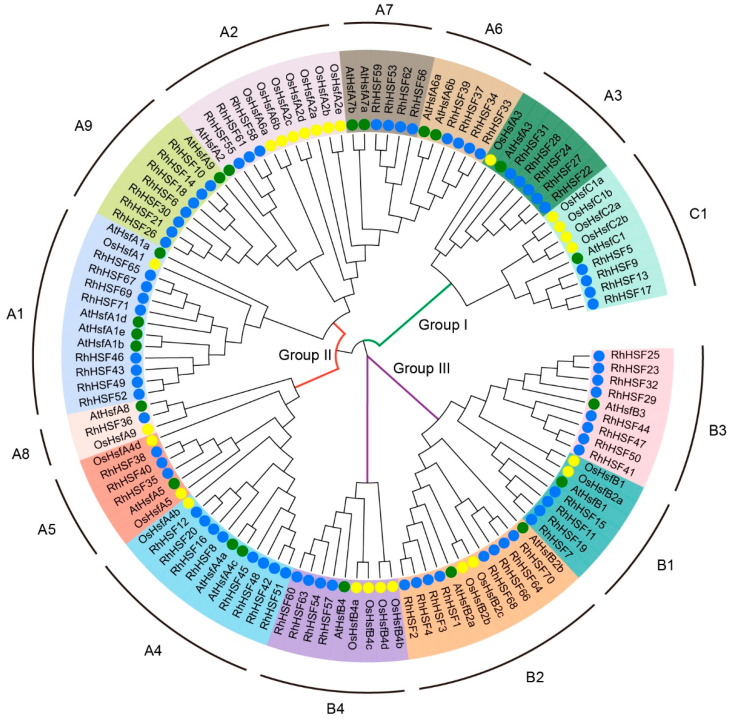
Phylogenetic analysis of HSF gene families in *Rosa hybrida*, *Arabidopsis thaliana*, and *Oryza sativa*. A maximum likelihood (ML) phylogenetic tree was constructed using 71 *R. hybrida* HSFs (RhHSFs), 21 *A. thaliana* HSFs (AtHSFs), and 25 *O. sativa* HSFs (OsHSFs). The tree was generated based on multiple sequence alignment of full-length HSF protein sequences with 1000 bootstrap replicates. Different colors indicate distinct subgroups (A1–A9, B1–B4, and C1) according to established HSF classifications. Groups I, II, and III represent the three major classes of plant HSFs. Colored circles denote species origin: blue for *R. hybrida*, yellow for *A. thaliana*, and green for *O. sativa*.

The *R. hybrida* HSF family was clearly divided into three major groups: Group I, Group II, and Group III (Figure 1). These groups were further subdivided into three subfamilies (HSFA, HSFB, and HSFC) based on the established classifications in *Arabidopsis* and rice, which correspond to the canonical HSF types. Group II encompassed all HSFA-type members, except HSFA3, comprising eight HSFA subfamilies (HSFA1 to HSFA9). HSFA members constituted the largest proportion of the RhHSF family and formed dominant branches in the phylogenetic tree (Figure 1). For instance, RhHSF46, RhHSF43, and RhHSF49 clustered closely with AtHSFA1a/b, suggesting conserved evolutionary lineages and potential functional orthology in heat stress response. Group I primarily included members of the HSFA3 and HSFC1 subfamilies (Figure 1). The close clustering of HSFA3 with HSFC1 could be due to shared functional roles or conserved domain structures, which is consistent with findings from other species, such as *Eriobotrya japonica* (loquat) and *Liriodendron chinense*, where similar relationships were reported. Group III was exclusively composed of HSFB members, which formed a well-supported clade distinct from the HSFA and HSFC groups, consistent with differences in domain architecture and functional roles (Figure 1). This group was further subdivided into three to four subfamilies (HSFB1–HSFB4), with HSFB1 and HSFB3 displaying close phylogenetic affinity, suggesting moderate conservation.

### 2.3. Conserved Motif Composition and Gene Structure of RhHSF Family

**Figure 2:**
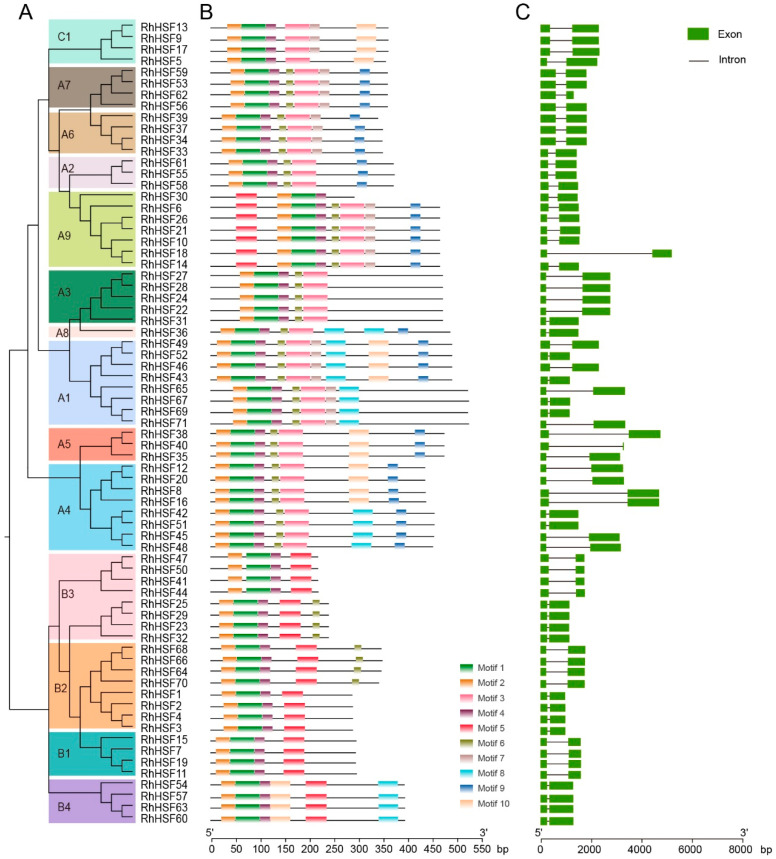
Phylogenetic relationships, conserved motif composition, and gene structures of HSF family members in *Rosa hybrida*. (**A**) Phylogenetic tree of 71 RhHSF proteins constructed using the maximum likelihood (ML) method with 1000 bootstrap replicates. Different colored blocks represent distinct subgroups (A1–A9, B1–B4, and C1). (**B**) Distribution of conserved motifs in RhHSF proteins identified by MEME. Ten motifs are shown in different colors, with motif numbers indicated. (**C**) Gene structures of *RhHSFs* showing exon–intron organization. Exons are represented by green boxes, and introns are indicated by black lines.

To gain a comprehensive understanding of the evolutionary dynamics and structural characteristics of the HSF gene family in *R. hybrida*, we conducted a systematic analysis of conserved motif composition and gene structure (exon–intron organization). Based on the phylogenetic tree construction, 71 RhHSF members were classified into 15 subfamilies (A1–A9, B1–B4, and C1), showing a high consistency with the evolutionary relationships illustrated in Figure 2A and Figure S1. Using the MEME suite, ten conserved motifs were identified across RhHSF proteins (Figure 2B and Figure S2). Among them, Motifs 1, 2, and 4 were universally present in all members, representing the highly conserved DNA-binding domain (DBD) that is responsible for recognizing heat shock elements (HSEs) and regulating downstream gene transcription. In contrast, distinct differences in motif composition and arrangement were observed among subgroups, suggesting structural and functional divergence within the family during evolution. Motif 3 was specifically enriched in HSFA and HSFC subgroups. The sequence characteristics of Motif 3—recurrent hydrophobic and charged residues—align with the structural features of HR-A/B regions within the oligomerization domain (OD), suggesting a role in trimeric complex formation. Additionally, Motif 9 was exclusively found in HSFA members, partially present in the A1 subgroup, completely absent in the A3 subgroup, and entirely absent from HSFB and HSFC subgroups. This motif is enriched in aromatic (F/Y/W), acidic (D/E), and hydrophobic (L/V/I) residues—hallmarks of the AHA-like activation domain—and is therefore inferred to form the core of the C-terminal transcriptional activation domain, which is essential for the transcriptional activator function of HSFAs. In comparison, Motif 5 was highly conserved in HSFB subgroup members, but nearly absent in class A and C members, except for occasional occurrence in the HSFA9 subgroup. This motif contains multiple charged residues and likely provides the structural basis for the repressor activity of HSFB proteins, consistent with previous functional findings such as GmHSFB2b, which directly binds target promoters to suppress gene expression [30].

Analysis of the exon–intron organization revealed notable structural variation among *RhHSF* genes (Figure 2C). All members contain 2 exons, indicating a relatively compact gene architecture across the family. However, certain genes, such as *RhHSF8* and *RhHSF16* (both classified within the HSFA4 subgroup), as well as *RhHSF18* (within the HSFA9 subgroup), and *RhHSF38* (within the HSFA5 subgroup), possess significantly extended intronic regions, implying potentially more intricate transcriptional regulation. In general, gene structure tends to be conserved within individual phylogenetic subgroups, reinforcing the clade classifications derived from phylogenetic analysis. This inter-subgroup variation in gene structure may highlight differences in regulatory complexity, splicing patterns, or expression dynamics among RhHSF classes.

### 2.4. Integrated Analysis of Cis-Element Distribution and Enrichment Patterns

**Figure 3:**
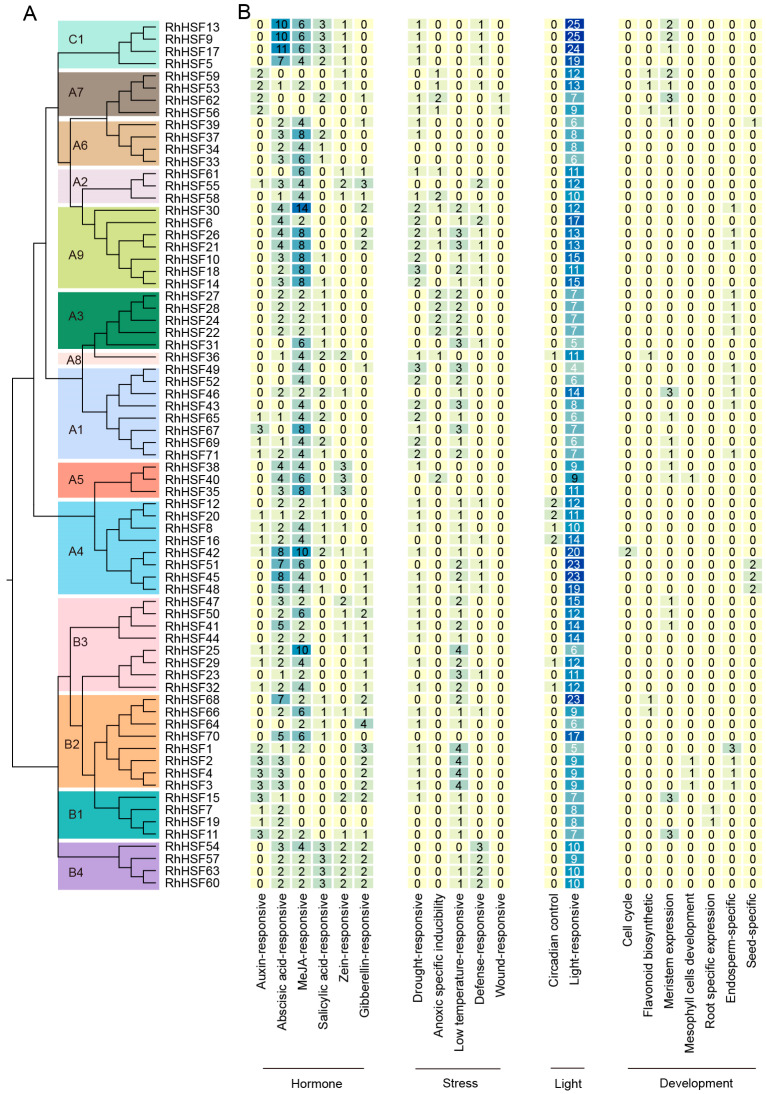
Cis-acting element analysis of *RhHSF* gene promoters. (**A**) Phylogenetic classification of RhHSFs into 15 subgroups (A1–A9, B1–B4, and C1) based on the full-length protein sequences. (**B**) Heatmap showing the frequency of cis-elements across individual *RhHSFs*. Darker blue indicates a higher number of elements, while lighter yellow represents fewer or none. Numerical values in each cell indicate the total number of each element type within each promoter region. Functional categories are labeled below: Hormone, Stress, Light, and Development.

To explore the upstream regulatory potential of *RhHSF* genes, a comprehensive analysis of cis-acting elements was conducted based on the 2 kb promoter regions (Table S3). The identified elements were functionally categorized into four major classes: hormone-responsive, stress-responsive, light-responsive, and development-related (Figure 3). Overall, although most *RhHSF* genes harbor only a limited number of cis-elements, clear subgroup-specific patterns emerged. Subgroups C1 and A4 exhibited the highest total number of cis-elements, predominantly enriched in light- and hormone-responsive elements, suggesting a more complex regulatory architecture (Figure 3A,B).

Among hormone-related elements, abscisic acid (ABRE) and jasmonic acid (CGTCA- and TGACG-motif) responsive elements were the most widespread across the family, while motifs associated with auxin and other hormones were relatively sparse (Figure 3A and Figure S3). Notably, C1, A4, and A9 subgroups showed specific enrichment of ABA and JA-responsive elements as illustrated in Figure 3B, supporting the hypothesis that these genes may function as regulatory nodes in hormone-mediated stress signaling. Similarly, B4 members, although lacking in auxin-responsive elements, contained nearly all other hormone-related motifs (Figure 3B), which suggests their broader involvement in hormonal regulatory networks. Stress-responsive elements, such as low-temperature responsive and drought-inducible motifs, were frequently detected, particularly in HSFA1, A9, and B2 subgroups (Figure 3B), highlighting their potential roles in abiotic stress adaptation. Light-responsive motifs were present in nearly all *RhHSFs* promoters, consistent with conserved photo regulatory functions reported for HSFs in other plant species [31,32]. Furthermore, developmental regulatory motifs, such as meristem- and seed-specific elements, were detected in only a few genes (e.g., *RhHSF15*, *RhHSF62, RhHSF45 and RhHSF51*) (Figure 3B), suggesting additional roles in tissue-specific expression or reproductive development. Taken together, these findings reveal that *RhHSFs* promoters exhibit modular and diversified cis-regulatory architectures, with subgroup-specific enrichment patterns that likely contribute to their distinct regulatory roles in hormonal signaling, environmental responsiveness, and developmental control.

### 2.5. Chromosomal Distribution and Synteny Analysis of RhHSFs

**Figure 4:**
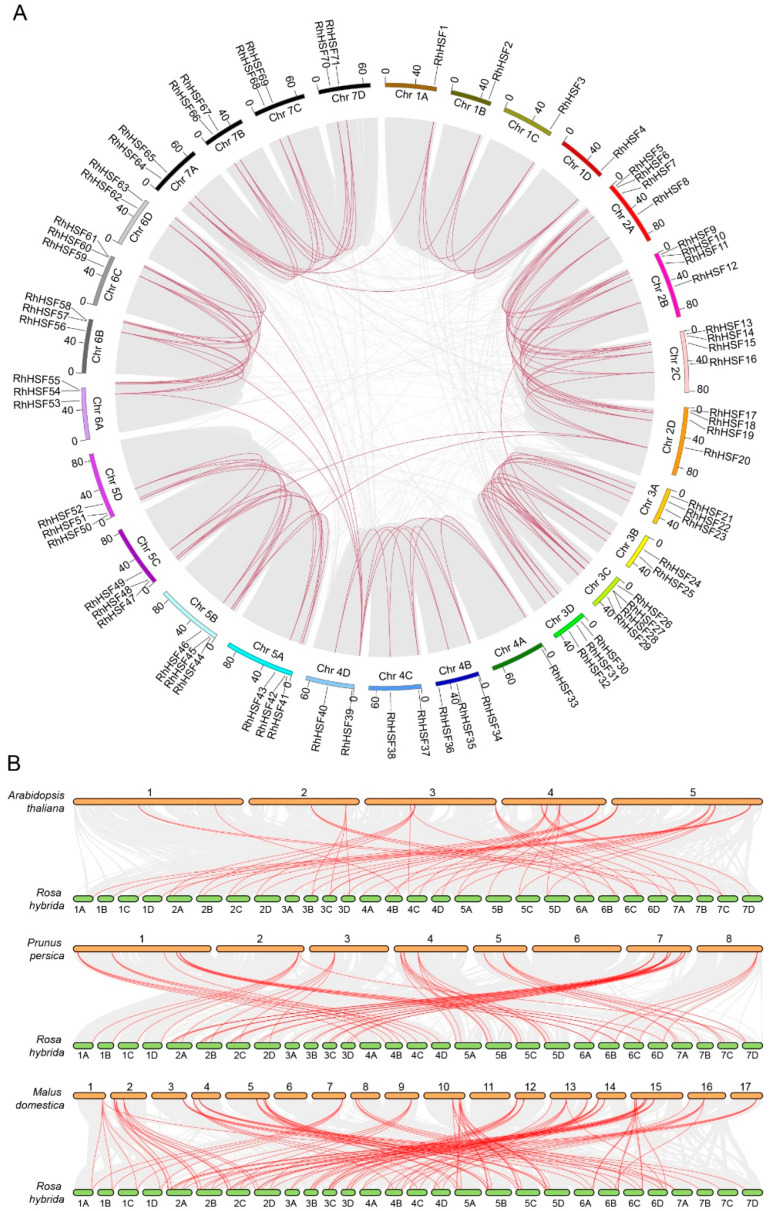
Chromosomal distribution and synteny analysis of HSF genes in *Rosa hybrida*. (**A**) Chromosomal mapping and intra-genomic synteny of 71 RhHSFs across 21 haplotype pseudo-chromosomes (Chr1A–Chr7D). Colored blocks indicate different chromosomes, and red curves represent segmental duplication gene pairs. (**B**) Comparative synteny analysis of RhHSFs with orthologous HSF genes in *Arabidopsis thaliana*, *Prunus persica*, and *Malus domestica*. Red lines represent conserved orthologous gene pairs between species.

To gain insight into the genomic organization and evolutionary expansion of the *RhHSF* gene family, we conducted chromosomal mapping, synteny analysis, and collinearity analysis on 71 gene members across 21 haplotype pseudo-chromosomes of *R. hybrida* (Figure 4A and Table S4). These genes were unevenly distributed, with notable gene clusters observed on chromosomes 2, 3, 5, and 6. Intra-species synteny analysis identified a total of 63 *RhHSF* gene pairs with high sequence homology. All *RhHSF* members were associated with whole-genome duplication (WGD) or segmental duplication, as evidenced by their localization within syntenic chromosomal regions. In contrast, no tandem duplications were detected, indicating that large-scale duplication events have served as the predominant evolutionary mechanism driving the expansion of the *RhHSF* gene family. The widespread distribution of segmental *RhHSF* duplicates across subgenomes is consistent with the paleopolyploid history and genomic complexity of modern rose.

Next, we conducted cross-species collinearity analysis between *R. hybrida* and three representative dicot species: *A. thaliana*, *Prunus persica* (*P. persica*), and *Malus domestica* (*M. domestica*) (Figure 4B and Table S5). A total of 50 orthologous HSF gene pairs were identified between *R. hybrida* and *A. thaliana*, 64 pairs with *P. persica*, and 114 pairs with *M. domestica*. These results indicate a closer evolutionary relationship between *Rosa* and *Malus*—both members of the *Rosaceae* family—while fewer conserved syntenic loci with *Arabidopsis*, a member of the *Brassicaceae*. Cross-species synteny analysis revealed a conserved subset of *RhHSF* genes collinear across all three species, including *RhHSF1*, *RhHSF2*, and *RhHSF3*, which are orthologous to *AT5G62020* (*A. thaliana*), *MdF0032103* (*M. domestica*), and *Prupe.2G292100* (*P. persica*), respectively. These highly conserved genes likely represent ancestral HSFs retained under strong purifying selection, suggesting functional constraint and regulatory conservation throughout angiosperm evolution. Conversely, several *RhHSF* members—particularly *RhHSF21*, *RhHSF26*, and *RhHSF30*, located on chromosomes 3 and 7—showed no detectable collinearity with any of the three reference species. These genes may have originated through lineage-specific duplication, or structural divergence, reflecting the dynamic evolutionary history and species-specific adaptation of the HSF gene family in *R. hybrida*.

### 2.6. Tissue-Specific Expression Profiles of RhHSFs

**Figure 5:**
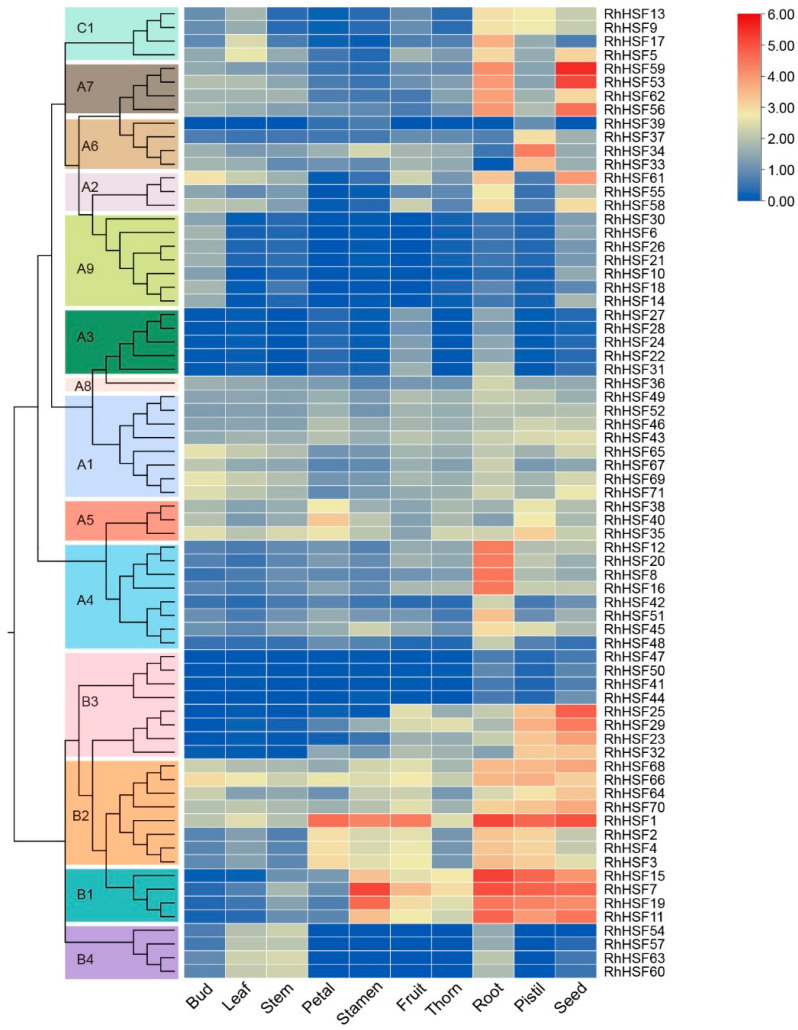
Tissue-specific expression profiles of *RhHSF* genes in *Rosa hybrida*. Hierarchical clustering heatmap of *RhHSF* genes across ten tissues: bud, leaf, stem, petal, stamen, fruit, thorn, root, pistil, and seed. Expression levels were calculated as log_2_(FPKM + 1) and normalized. The expression levels are represented by the color scale (blue to red), where blue indicates low expression and red indicates high expression. Genes are grouped according to their phylogenetic subfamilies (A1–A9, B1–B4, C1), as indicated by the colored bars on the left.

To explore the potential biological roles of *RhHSF* genes, their transcriptional profiles were analyzed across ten tissues of *R. hybrida*, including bud, leaf, stem, petal, stamen, fruit, thorn, root, pistil, and seed (Figure 5 and Table S6). The expression patterns revealed pronounced tissue specificity among different *RhHSF* members. As shown in Figure 5, members of the HSFA1, A5, A8, and B2 subgroups exhibited relatively broad expression across the ten surveyed tissues, suggesting their involvement in fundamental aspects of plant growth and development. In contrast, other subgroups displayed more tissue-specific expression patterns. For instance, HSFB1 genes (e.g., *RhHSF15* and *RhHSF19*) showed markedly higher expression in reproductive organs, particularly stamen, pistil, fruit, and seed, pointing to potential roles in floral organ development and reproductive regulation. Members of the B4 subgroup were predominantly expressed in vegetative organs such as root, stem, and leaf, implying functions associated with general plant growth. Interestingly, most subgroups also showed relatively elevated expression in roots, highlighting a possible contribution of RhHSFs to root developmental or stress-related processes.

Notably, A7 subgroup genes, including *RhHSF53* and *RhHSF59*, exhibited sharp expression peaks specifically in seeds, suggesting specialized functions during seed maturation or reproduction. In contrast, several B3 subgroup members displayed consistently low expression across all ten tissues, which may reflect either tissue-restricted expression not captured in this dataset, or functions activated only under specific stress or developmental cues. Collectively, these results indicate that *RhHSF* genes exhibit both ubiquitous and organ-specific expression patterns, highlighting their likely diversified roles in developmental regulation and tissue-specific stress responsiveness in rose.

### 2.7. Dynamics of RhHSF Gene Expression Under Heat Stress

**Figure 6:**
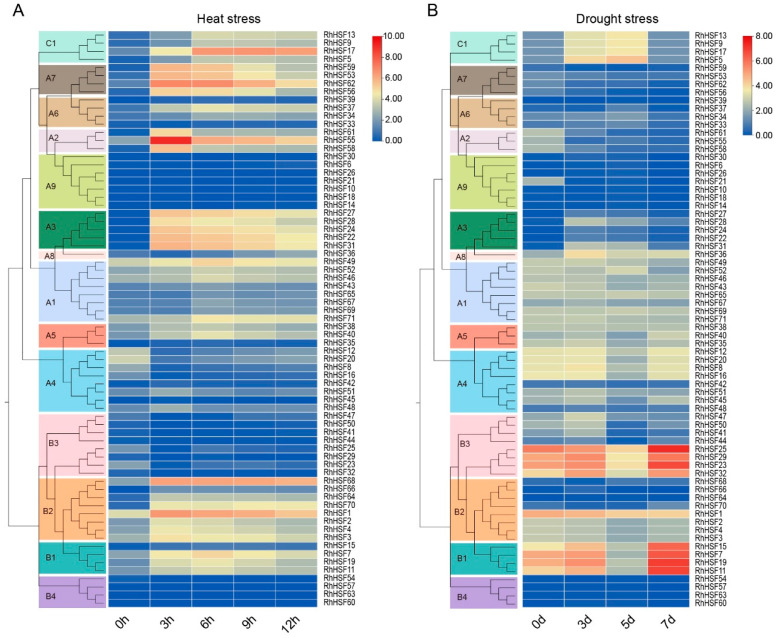
Expression profiles of HSF genes in *Rosa hybrida* under heat and drought stress. (**A**) Hierarchical clustering heatmap of *RhHSF* genes in response to heat stress (42 °C) at 0 h, 3 h, 6 h, 9 h, and 12 h. (**B**) Hierarchical clustering heatmap of *RhHSF* genes in response to drought stress at 0, 3, 5, and 7 days after water withholding. Expression levels were calculated as log_2_(FPKM + 1) and normalized. The color scale represents relative expression levels, with blue indicating low expression and red indicating high expression. Genes are clustered according to their phylogenetic subfamilies (A1–A9, B1–B4, C1), as shown on the left.

To investigate the responsiveness of *RhHSF* genes to high-temperature stress, transcriptome data were analyzed across five time points (0 h, 3 h, 6 h, 9 h, and 12 h) following 42 °C heat treatment in *R. hybrida* (Figure 6A and Table S7). With the exception of subgroups A4, A9, B3, and B4, most RhHSF members exhibited an overall upward trend under heat shock conditions and were differentially regulated in a time-dependent manner, suggesting their involvement in orchestrating heat stress responses. Members of the A2, A3, A7, and B2 subgroups showed early and transient upregulation, with expression peaking at 3 h and subsequently declining. This pattern indicates that these genes may participate in the initial perception of heat stress or in rapid signaling cascades. Notably, *RhHSF55* displayed an exceptionally sharp induction, increasing more than 130-fold within the first 3 h (Table S7), reinforcing the hypothesis that it acts as a pivotal early-phase modulator of heat-responsive pathways.

In contrast, some genes from the A1, A5, and B1 subgroups (e.g., *RhHSF7*, *RhHSF49*) reached their maximum expression at 6 h, followed by a decline (Figure 6A). However, their overall induction levels remained relatively modest, suggesting a more moderate role in the heat stress response. Members of the HSFC1 subgroup, including *RhHSF5*, *RhHSF9*, and *RhHSF17*, exhibited gradual and sustained upregulation, with expression peaking at 9 h (Figure 6A and Table S7), implying that these genes function as mid-to-late response regulators during prolonged heat exposure. Conversely, most A9, B3, and B4 members remained at low or undetectable levels during heat treatment, suggesting minimal contribution to heat stress adaptation.

### 2.8. Expression Profiles of RhHSFs Under Drought Stress

To elucidate the transcriptional responses of *RhHSF* genes to drought stress, expression profiles were analyzed at 0, 3, 5, and 7 days after water withholding in 60-day-old *R. hybrida* ‘Samantha’ seedlings (Figure 6B and Table S8). During drought stress, the majority of HSFA subfamily genes (A1–A9) exhibited relatively stable expression levels, suggesting a lack of direct involvement in drought responsiveness. However, genes in the A3 and A8 subgroups showed a transient induction at day 3 that declined thereafter, consistent with a role in the intermediate phase of the response. By contrast, several A2 members and one A9 gene (*RhHSF21*) exhibited a slight decrease at day 3, which may represent early suppression rather than activation.

The strongest induction was observed in the B1 and B3 subgroups. For example, *RhHSF11* and *RhHSF19* exhibited a clear time-dependent upregulation, with transcript levels peaking at day 7 and increasing more than fivefold relative to baseline (Figure 6B and Table S8). These findings indicate that B1 and B3 members act as late-stage regulators of drought adaptation. In addition, members of the C1 subgroup (e.g., *RhHSF9*, *RhHSF13*, *RhHSF17*) exhibited distinct early-to-mid responses, showing sharp increases at day 3 or 5 that declined by day 7, implying a transient regulatory role in the early drought response.

### 2.9. Protein–Protein Interaction Network Analysis of RhHSFs

**Figure 7:**
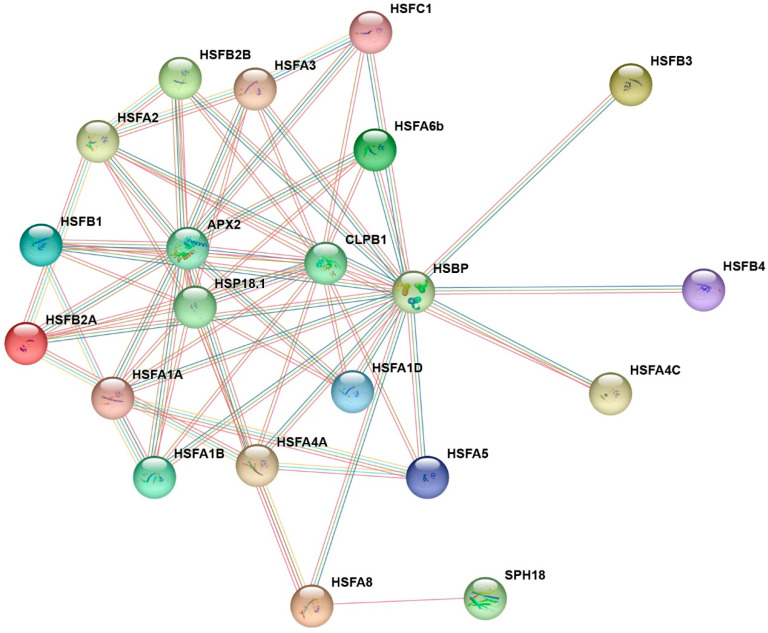
Predicted protein–protein interaction (PPI) network of HSF proteins in *Rosa hybrida*. The PPI network was constructed based on orthologous interactions in *Arabidopsis thaliana* using the STRING database with a high confidence score (≥0.7). Nodes represent proteins, and edges indicate predicted functional associations. Core hub proteins show high connectivity, highlighting their potential central roles in the heat shock regulatory network.

To gain insights into the functional landscape of RhHSF proteins, we constructed a protein–protein interaction (PPI) network based on orthologous interactions from *A. thaliana* (Figure 7 and Tables S9 and S10). The inferred network revealed that core regulators—such as HSFA1A (RhHSF41, RhHSF44, RhHSF50, RhHSF67), HSFA1B (RhHSF43, RhHSF45), and HSFB2A (RhHSF55)—exhibited high connectivity (Figure 7), suggesting their central roles in orchestrating heat stress responses. HSBP emerged as a prominent hub protein, interacting with multiple HSFs including HSFA1A and HSFA5, in line with its established role as a negative modulator of HSF activation. Additionally, ASCORBATE PEROXIDASE 2 (APX2), a ROS-scavenging enzyme, and Caseinolytic Peptidase B1 (CLPB1), a heat-induced chaperone, were found to interact with multiple HSFA members. Specifically, APX2 was associated with HSFA1A, HSFA1B, HSFA2, and HSFA4A, suggesting its involvement in oxidative stress mitigation via HSF-regulated pathways. CLPB1 exhibited broader interactions with six HSFA proteins, including HSFA1A, HSFA1B, HSFA1D, HSFA2, HSFA4A, and HSFA5, indicating its cooperative role in maintaining protein homeostasis during thermal stress. In contrast, HSFs like HSFB3 (RhHSF59) and HSFA4C (RhHSF50) displayed limited interaction profiles, suggesting more specialized, possibly stimulus- or tissue-specific regulatory roles within the broader stress response network.

### 2.10. The Functional Analysis of RhHSF17

**Figure 8:**
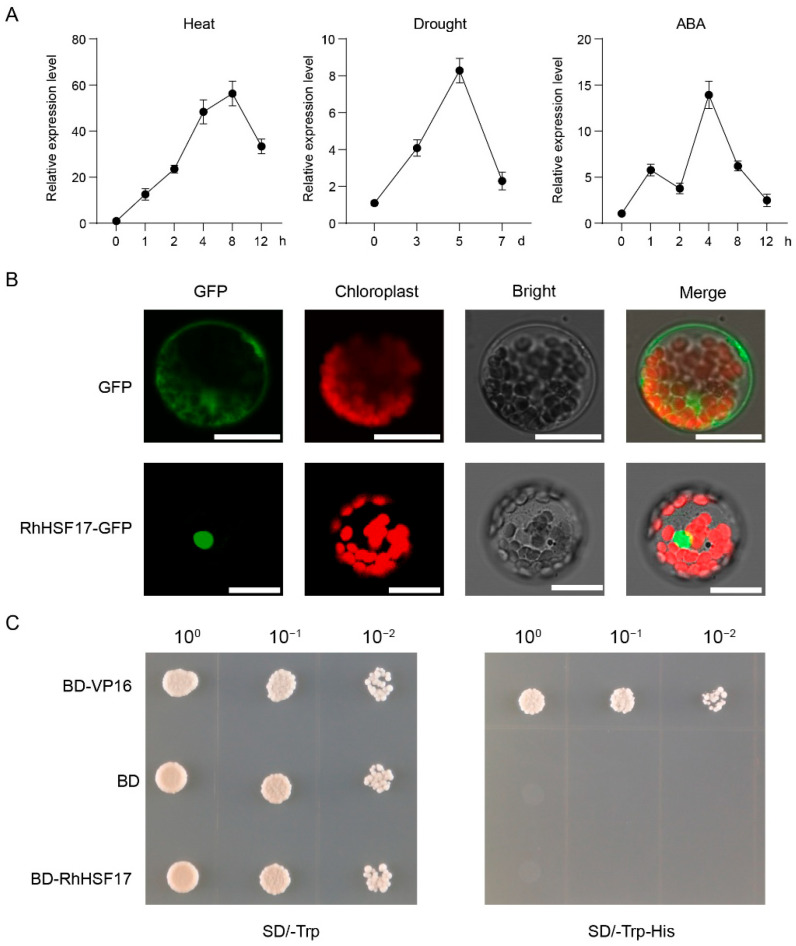
Functional investigation of *RhHSF17* in *Rosa hybrida*. (**A**) Quantitative real-time PCR (qRT-PCR) analysis of *RhHSF17* expression under heat (42 °C), drought, and abscisic acid (ABA, 100 μM) treatments. Relative expression levels were normalized to the reference gene *RhUBI2* and calculated using the 2^−ΔΔCt^ method. Error bars represent the mean ± standard deviation (SD) of three biological replicates. (**B**) Subcellular localization of RhHSF17-GFP fusion protein in *Arabidopsis thaliana* mesophyll protoplasts. GFP alone (top row) shows ubiquitous fluorescence, while RhHSF17-GFP (bottom row) localizes specifically to the nucleus. GFP signals (green), chloroplast autofluorescence (red), bright-field images, and merged views are shown. Scale bars = 20 μm. (**C**) Transcriptional activation assay of RhHSF17 in yeast. Yeast cells expressing BD-RhHSF17, BD-VP16 (positive control), or BD (negative control) were cultured on SD/-Trp (left) and SD/-Trp-His (right) media under threefold serial dilutions (10^0^, 10^−1^, 10^−2^). BD-RhHSF17 showed no growth on selective medium, implying no transcriptional activation in yeast.

Transcriptome profiling under both heat and drought stress conditions revealed that members of the HSFC subfamily were transcriptionally responsive to both stimuli, suggesting their potential involvement in abiotic stress signaling. Among them, *RhHSF17* exhibited consistent and pronounced upregulation across both treatments and was therefore selected for further functional characterization. To validate the transcriptome findings, we performed qRT-PCR analysis of *RhHSF17* expression under heat and drought treatments. The results confirmed that *RhHSF17* was significantly upregulated by both stresses, consistent with the RNA-seq data (Figure 8A). In addition, we examined its response to abscisic acid (ABA) treatment and found that *RhHSF17* expression was markedly induced by 100 μM ABA, reaching its peak at 4 h post-treatment (Figure 8A). This strong ABA responsiveness suggests that *RhHSF17* may function as a downstream target or component of ABA-mediated stress signaling pathways. To determine its subcellular localization, a RhHSF17-GFP fusion construct was transiently expressed in *A. thaliana* mesophyll protoplasts. As shown in Figure 8B, the control GFP signal was distributed throughout the cytoplasm and nucleus, whereas RhHSF17-GFP fluorescence was strictly confined to the nucleus, overlapping with the chloroplast autofluorescence in merged images. These results confirm that RhHSF17 is a nuclear-localized protein, consistent with its predicted role as a transcription factor. To assess its transcriptional activity, RhHSF17 was fused to the GAL4 DNA-binding domain (BD) and expressed in yeast cells. As shown in Figure 8C, yeast cells expressing BD-RhHSF17 did not grow on SD medium lacking tryptophan and histidine (SD/-Trp-His), similar to the empty vector control, whereas the positive control BD-VP16 exhibited robust growth. These results indicate that RhHSF17 lacks intrinsic transcriptional activation activity in yeast. This suggests that RhHSF17 may function through interactions with other transcriptional regulators, such as class A HSFs, to modulate stress-responsive gene expression in *R. hybrida*.

### 2.11. RhHSF17 Overexpression Enhances Drought Tolerance in Arabidopsis

**Figure 9:**
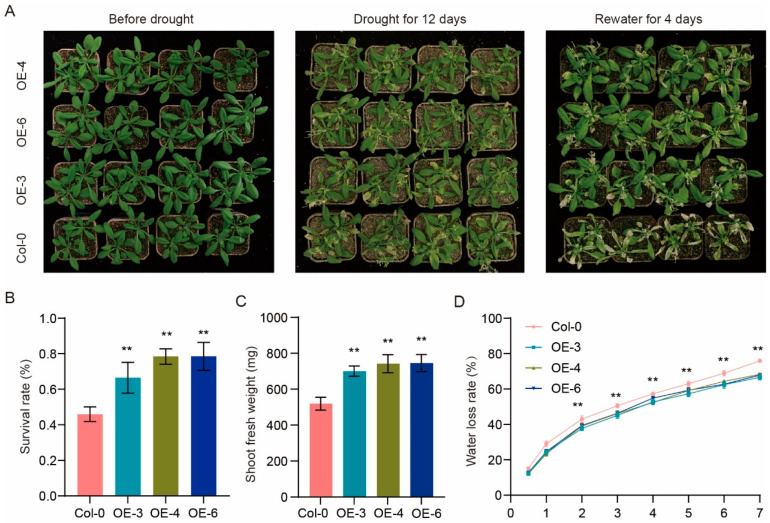
*RhHSF17* overexpression enhances drought tolerance in Arabidopsis. (**A**) Phenotypic comparison of wild-type (Col-0) and three independent *RhHSF17*-overexpressing Arabidopsis lines (OE-3, OE-4, OE-6) under drought treatment. Plants were photographed before drought, after 12 days of drought stress, and after 4 days of rewatering. (**B**) Survival rate of Col-0 and *RhHSF17*-overexpressing lines following drought treatment and rewatering. (**C**) Shoot fresh weight of Col-0 and *RhHSF17*-overexpressing lines under drought stress. (**D**) Water loss rate of detached rosette leaves from Col-0 and *RhHSF17*-overexpressing lines during dehydration for 7 h. Data represent means ± SD of three biological replicates. Asterisks indicate statistically significant differences compared with Col-0 (** *p* < 0.01; Student’s *t*-test).

To further evaluate the role of *RhHSF17* in abiotic stress adaptation, we examined the drought response of transgenic Arabidopsis lines. Under normal growth conditions, no obvious differences in morphology or biomass were observed between Col-0 and *RhHSF17*-overexpressing plants (OE-3, OE-4, OE-6). However, after 12 days of water withholding, Col-0 plants displayed severe wilting, while *RhHSF17*-overexpressing lines maintained greener and more turgid leaves. Upon rewatering, transgenic plants exhibited a markedly higher recovery rate compared with Col-0 (Figure 9A). Quantitative analysis confirmed these phenotypic differences. The survival rate of transgenic lines was significantly higher than that of Col-0 (Figure 9B). Similarly, shoot fresh weight was increased in *RhHSF17*-overexpressing plants under drought stress (Figure 9C). In addition, detached leaf assays showed that the water loss rate was consistently lower in the transgenic lines than in Col-0 (Figure 9D), suggesting improved water retention capacity. Together, these results demonstrate that *RhHSF17* positively regulates drought tolerance in Arabidopsis, likely by enhancing water-use efficiency and maintaining cellular homeostasis under dehydration stress.

## 3. Discussion

Heat shock transcription factors (HSFs) are central regulators that play key roles in enhancing plant thermotolerance by regulating the expression of heat shock proteins (HSPs) [11]. In addition to heat stress, HSFs have also been implicated in responses to other abiotic stresses, such as drought and salinity [9]. *R. hybrida* is a temperature-sensitive ornamental species that blooms optimally at 22–26 °C, while high temperatures reduce flower size and number or even inhibit blooming by inducing floral dormancy [28,33,34]. Recent studies have highlighted the important roles of HSFs in the heat stress response of cultivated roses. However, due to the recent release of the tetraploid *R. hybrida* genome [29], a comprehensive analysis of the HSF gene family in rose is still lacking. In this study, we identified 71 HSF family members from the pseudo-chromosomes of the tetraploid rose ‘Samantha’ haplotype genome. Although previous studies have reported 19 HSF genes in diploid *Rosa chinensis* [23], the tetraploid ‘Samantha’ genome yielded only 71 members—slightly fewer than the expected expansion (~76 members). This likely reflects post-polyploid gene loss and fractionation—a common outcome of whole-genome duplication (WGD)—where redundant copies are eliminated or functionally diverge through sub-functionalization or pseudogenization [35]. Our further analysis revealed that the HSF gene family in modern *Rosa* has undergone extensive expansion primarily through segmental duplication, with no evidence of tandem duplication observed. A similar pattern has been reported in *Camellia sinensis* and garlic [31,36], suggesting that segmental duplication may be the predominant driving force in the evolutionary expansion of the HSF gene family. In angiosperms, the HSF gene family has undergone extensive duplications, resulting in a diverse repertoire of members with distinct structural and functional specializations [37]. Our phylogenetic analysis grouped the 71 RhHSF proteins from cultivated *R. hybrida* into three major classes—A, B, and C—consistent with the typical classification established in Arabidopsis. Conserved motif analysis further highlights structural divergence among RhHSF subfamilies. For example, motif 3, found exclusively in HSFA and HSFC members, aligns with the hydrophobic HR-A/B region of the oligomerization domain, suggesting a role in trimer formation—a prerequisite for HSF activation and DNA binding under stress conditions [10]. Moreover, the higher similarity of this motif between classes A and C, compared to class B, may partly explain the closer phylogenetic relationship observed between HSFA and HSFC members.

Among the four major categories of cis-elements, stress- and hormone-responsive motifs were the most abundant, while development-related elements were comparatively rare. This enrichment pattern suggests that *RhHSFs* function mainly in environmental signal perception and hormone-mediated transcriptional regulation. Within the hormone-responsive class, abscisic acid (ABA)-related elements (e.g., ABRE) and jasmonic acid (JA)-related elements (e.g., CGTCA-motif, TGACG-motif) were the most abundant across *RhHSF* promoters. These motifs were particularly enriched in the *C1*, *A4*, and *A9* subgroups, suggesting that genes within these clades may serve as key transcriptional integrators of ABA and JA signaling under stress conditions in *R. hybrida*. ABA is a well-established master regulator of abiotic stress tolerance—especially under drought and salinity—and also plays critical roles in seed dormancy and germination [38,39]. Functional studies in *chrysanthemum* have demonstrated that the ABA-responsive *CmHSFA4* gene in enhances salt stress tolerance via ABA-mediated pathways [40]. Moreover, ABA-INSENSITIVE3 (ABI3) has been shown to directly activate the *AtHSFA9* promoter through ABA signaling, linking HSF-mediated transcription to seed maturation [41]. In the context of stress-specific motif analysis, the significant enrichment of drought- and low-temperature-responsive elements within the A1, A9, and C1 subgroups suggests that these genes are integral to the transcriptional reprogramming underlying abiotic stress acclimation. Such low-temperature-responsive elements may also be linked to JA signaling, as heat shock-induced cold acclimation in cucumber has been shown to involve CsHSFA1d-mediated activation of JA biosynthesis and signaling [42]. This motif enrichment aligns with our expression data, where A1 and C1 members exhibited transient modulation under both heat and drought stress, reflecting their dynamic and context-dependent regulation during stress responses. Moreover, the broad occurrence of light-responsive elements across nearly all *RhHSF* promoters reflects their potential conservation in photomorphogenic regulation, possibly linking circadian rhythms and thermotolerance. Consistent with this, previous studies showed that thermotolerance in Arabidopsis exhibits a distinct daytime rhythm linked to light signaling inputs [43].

The spatial and temporal expression patterns of *RhHSFs* across tissues and abiotic stresses highlight a highly specialized transcriptional network in *R. hybrida*. The tissue-specific profiles indicate that distinct subgroups are tuned to particular developmental programs. *HSFA1* and *HSFA5* are broadly expressed across all ten tissues, with relatively high expression in leaves, stems, and roots, supporting their roles as key regulators of basal protection and vegetative growth. In contrast, HSFB1 and B2 subgroups showed pronounced expression in stamens and pistils, similar to reports in tomato and wheat, where HSFB genes contribute to reproductive organ development and thermotolerance. Notably, the seed-specific expression of the HSFA7 subgroup in rose resembles the expression pattern observed in rice [44]. This divergence hints at a possible neo-functionalization of HSF genes in rosa, reflecting the co-evolution of stress signaling networks.

Our heat stress time-course analysis revealed that A2, A3, and A7 subgroup members were rapidly induced within the first 3–6 h of heat treatment, consistent with the known roles of A-class HSFs as transcriptional activators. In our data, *RhHSF55* (A2) showed a sharp and transient peak at 3 h, indicating its early involvement in triggering downstream heat shock proteins (HSPs) networks. Such early and transient activation is consistent with the canonical role of HSFA2 and A3 orthologs in other species, which act as rapid-response transcriptional activators [45,46,47]. Of particular note is the gradual and sustained induction of C1 subgroup genes, which reached peak expression between 6 and 9 h. Unlike in rice, where *OsHSFC1a* is down-regulated by early heat stress [48], and Arabidopsis, where HSFC1 functions primarily as a co-regulator rather than a late-phase activator, the rose C1 members may have acquired a specialized role in sustaining heat stress responses, potentially enabling prolonged thermotolerance or transcriptional memory in ornamental species. Unlike the pronounced heat response, drought stress induced fewer but more subgroup-specific transcriptional changes, with notable activation of C1, B1 and B3 genes. This subgroup-specific activation aligns with recent evidence that HSFB members not only act as co-repressors but can also integrate cross-talk between drought and heat stress pathways [49]. The early-to-mid induction of C1 members (day 3–5) further underscores their regulatory flexibility, suggesting a transient role in sensing or modulating drought-related transcriptional shifts. Conversely, the overall low responsiveness of A-group genes to drought contrasts with studies in Arabidopsis and soybean, where A1 and A2 are strongly induced under dehydration [15]. This discrepancy may reflect species-specific adaptations or the unique physiological traits of rose, which has a complex hybrid genome and specialized water management strategies in its ornamental organs.

Heat and drought stresses often co-occur, leading to compounded detrimental effects on plant growth and productivity, as both stresses disrupt cellular homeostasis, impair photosynthesis, and induce oxidative stress through overlapping signaling pathways [50]. The PPI network analysis highlights APX2, a key ROS scavenger, as an important interactor of RhHSFs, suggesting that these factors may operate within a tightly coordinated ROS-protein homeostasis regulatory axis [9]. Transcriptome data further revealed that, among different HSF classes, class C HSFs exhibited the most prominent responses under combined heat and drought stress. Notably, most RhHSFC subgroup members contain ABA-responsive cis-elements, implying that they may participate in cross-talk between heat/drought stress signaling and the ABA pathway. To investigate this possibility, we selected *RhHSF17*, a representative C1 subgroup gene, for preliminary functional validation. Expression analysis showed that *RhHSF17* is strongly induced by heat, drought, and ABA treatments, and subcellular localization confirmed its nuclear localization, consistent with its role as a transcription factor. However, transcriptional activation assays indicated that RhHSF17 lacks autonomous transactivation activity. Given that HSFA-type HSFs act as the master regulators of heat stress response (HSR) [19], it is plausible that RhHSF17 may cooperate with A-class HSFs to form heteromeric complexes, coordinating transcriptional responses to combined heat and drought stress in *R. hybrida*. This hypothesis is supported by studies in wheat showing that TaHsfA2h, acting as a co-activator, interacts with TaHsfC2a to activate ABA and ROS signaling pathways under heat stress [51]. Similarly, HvHSFA2e has been reported to enhance drought and heat tolerance by upregulating heat-responsive genes and modulating ABA and flavonoid biosynthesis pathways [22]. These observations suggests that RhHSF17 may function primarily as a co-regulator, exerting its function through protein–protein interactions with activator-type HSFs rather than by independently initiating transcription. Unlike class A and B HSFs, the role of class C HSFs in plant thermotolerance remains poorly characterized, though recent studies point toward their importance in abiotic stress adaptation. In wheat (*Triticum aestivum*), for example, overexpression of *HSFC2a* enhances heat tolerance during grain development via the ABA signaling pathway by binding to heat stress elements (HSEs) in promoters of downstream target genes, including HSPs and galactinol synthase [52]. Similarly, in Amur grape (*Vitis amurensis*), *VaHSFC1* confers tolerance to multiple abiotic stresses such as heat, cold, and salinity, and its overexpression in *Arabidopsis* enhances thermotolerance by upregulating APX2 and HSP expression, improving membrane stability, and increasing chlorophyll content, although these transgenic plants also exhibit hypersensitivity to ABA [53]. Our findings, although preliminary, highlight the potential importance of C-class HSFs, especially *RhHSF17*, in the stress responses of *R. hybrida*. The sustained induction of *RhHSF17* under combined heat and drought stress, together with its ABA responsiveness, suggests that C-class HSFs may help maintain transcriptional activity when early A- and B-class responses decline. Given that cultivated roses are sensitive to heat and drought, which can directly affect flower quality and longevity, understanding the function of C-class HSFs could provide new insights for breeding stress-resilient cultivars. Future studies on RhHSF17 and other C-class members may offer practical strategies to improve thermotolerance and drought adaptability without compromising ornamental traits.

## 4. Materials and Methods

### 4.1. Genome-Wide Identification of HSF Genes in Rosa hybrida

The tetraploid *R. hybrida* cv. ‘Samantha’ genome and corresponding annotation files were retrieved from the Rosa genome database (https://doi.org/10.6084/m9.figshare.22774097) [29]. To identify putative heat shock transcription factor (HSF) genes, a Hidden Markov Model (HMM) profile of the HSF-specific DNA-binding domain (DBD; PF00447) was obtained from the Pfam database (currently hosted by InterPro) (http://pfam.xfam.org/, accessed on 2 November 2024) and used as a query to perform an HMMER search (v3.3) against the ‘Samantha’ protein database, with an E-value cutoff of 1 × 10^−5^.

Candidate HSF proteins were further validated using multiple domain analysis tools, including the NCBI Conserved Domain Database (CDD, https://www.ncbi.nlm.nih.gov/Structure/cdd/, accessed on 10 November 2024), Pfam (http://pfam.xfam.org/, accessed on 10 November 2024), and SMART (http://smart.embl.de/, accessed on 10 November 2024), to confirm the presence of canonical HSF domains. To refine gene identification, a BLASTP search was also performed against the *A. thaliana* HSF protein database obtained from TAIR (The Arabidopsis Information Resource) (https://www.arabidopsis.org, accessed on 10 November 2024), using a stringent E-value threshold of 1 × 10^−10^. Sequences lacking conserved domains or showing poor homology were excluded. Subsequently, the gene structures of remaining candidates were manually curated based on reference genome annotations and further corrected using transcriptome assembly data from ‘Samantha’.

The isoelectric point (pI) and molecular weight of predicted HSF genes were estimated via ExPASy ProtParam (https://www.expasy.org/; accessed on 11 November 2024). Subcellular localization of RhHSFs was inferred using WoLF PSORT tools (https://wolfpsort.hgc.jp/; accessed on 11 November 2024).

### 4.2. Phylogenetic Analysis and Classification of RhHSF Genes

To investigate the evolutionary relationships and subfamily classification of heat shock transcription factors (HSFs) in *R. hybrida*, a total of 71 full-length HSF protein sequences from *R. hybrida* were aligned together with representative HSF proteins from *A. thaliana* (21) and *O. sativa* (25). Multiple sequence alignment was performed using Clustal Omega (v1.2.4) with default parameters to ensure high-quality global alignment of conserved domains and divergent regions. The aligned sequences were then used to construct a phylogenetic tree using the Maximum Likelihood (ML) method implemented in MEGA X [54]. The reliability of the branching was evaluated using 1000 bootstrap replicates, and evolutionary distances were calculated based on the Jones–Taylor–Thornton (JTT) substitution model. Based on the topological structure of the resulting phylogenetic tree and prior classification criteria established in Arabidopsis and rice. The resulting phylogenetic tree was further edited and visualized using the EvolView online platform (https://evolgenius.info/evolview-v2/, accessed on 20 November 2024) [55].

### 4.3. Conserved Motif and Gene Structure Analysis

Conserved motifs among RhHSF proteins were identified using the Multiple Em for Motif Elicitation (MEME) Suite (v5.0.5). The parameters were configured to identify up to 10 distinct motifs, with motif widths set between 6 and 50 amino acids, and the maximum number of motifs per sequence limited to 10. Motifs with statistically significant E-values were retained for further analysis. Gene structures, including exon–intron organization, by aligning each HSF coding sequence (CDS) with its corresponding genomic DNA sequence. The gene models were visualized using the Gene Structure Display Server (GSDS 2.0).

### 4.4. Cis-Acting Element Prediction in Promoters

To analyze the regulatory elements in promoter regions, 2 kb upstream sequences from the transcription start site of each *RhHSF* gene were extracted from the tetraploid *Rosa hybrida* ‘Samantha’ genome sequences. These sequences were then submitted to the PlantCARE database (http://bioinformatics.psb.ugent.be/webtools/plantcare/html/; accessed on 11 December 2024) to identify cis-acting elements. All predicted elements were manually curated and subsequently classified into four major functional categories—hormone-responsive, stress-responsive, light-responsive, and development-related—based on their documented roles in plant physiological and developmental processes. The distribution of different cis-acting elements within the 2 kb upstream promoter regions was visualized using TBtools II software (v2.309, https://github.com/CJ-Chen/TBtools-II/releases, accessed on 16 December 2024). Heatmaps representing the abundance and enrichment patterns of various cis-elements were generated using ggplot2 (v3.4.4) in the R programming (v4.1.2).

### 4.5. Chromosomal Mapping and Synteny Analysis

The genomic distribution and duplication patterns of *RhHSF* genes were investigated through intra- and inter-species synteny analyses. Intra-genomic synteny analysis was performed using MCScanX [56], which detects collinear gene pairs based on pairwise comparisons of all protein-coding genes within the *R. hybrida* ‘Samantha’ genome. Prior to analysis, an all-against-all BLASTP search was conducted with an E-value cutoff of 1 × 10^−5^ to generate the input file required by MCScanX. Gene duplication events were classified into segmental, tandem, proximal, and dispersed duplications according to the MCScanX output classification scheme. The genomic positions of *RhHSF* genes were inferred from the intra-genomic collinearity analysis and visualized using a Circos diagram. This diagram illustrates the gene relationships within the *R. hybrida* genome, showing the relative locations of *RhHSF* genes on pseudo-chromosomes based on the collinear segmental duplication relationships. In addition, cross-species collinearity analyses between *R. hybrida*, *A. thaliana*, *P. persica*, and *M. domestica* were also conducted using MCScanX, and the results were visualized using Circos (v0.69), which shows the syntenic relationships between *R. hybrida* and the other species.

### 4.6. RNA-Seq Data Processing and Expression Profiling

To characterize the expression patterns of *RhHSFs* across different tissues and under heat stress, RNA-seq raw data were obtained from the NCBI BioProject database. Tissue-specific transcriptome datasets covering ten organs—bud, leaf, stem, petal, stamen, fruit, thorn, root, pistil, and seed—were retrieved under the accession number PRJNA1108167 [29]. Heat stress-related RNA-seq data, derived from *Rosa chinensis* seedlings exposed to 42 °C for 0, 3, 6, 9, and 12 h, were downloaded from PRJNA1090540 [27]. For the drought stress experiment, Ninety-day-old ‘Samantha’ seedlings were subjected to water withholding, and leaf samples were collected at 0, 3, 5, and 7 days after drought onset. These data were deposited under BioProject accession number PRJNA1295141. For both heat and drought stress treatments, three biological replicates were used per condition, with each replicate representing three independent seedlings.

All raw reads were subjected to quality control using FastQC (v0.11.9) and trimmed with Trimmomatic (v0.39) to remove low-quality bases and adapters. Clean reads were then aligned to the tetraploid *R. hybrida* ‘Samantha’ reference genome using HISAT2 (v2.2.1) with default settings [57]. Following alignment, transcript assembly and quantification were performed using StringTie (v2.1.4) [58]. Expression levels of *RhHSFs* were calculated as FPKM (Fragments Per Kilobase of transcript per Million mapped reads) to account for gene length and sequencing depth. Expression values were normalized using the transformation log_2_ (FPKM + 1) prior to visualization. Heatmaps representing tissue-specific and heat stress responsive expression patterns were generated using TBtools II [59]. For RNA-seq analyses, the primary goal was to profile gene expression, and no multiple testing correction was applied.

### 4.7. Protein–Protein Interaction Network Construction

To infer protein–protein interactions (PPIs), Arabidopsis homologs of RhHSF proteins were identified using BLASTP. The Arabidopsis interaction data were retrieved from the STRING database (v11.5) with a confidence score ≥ 0.7. The PPI network was visualized using Cytoscape (v3.9.1), and hub genes were identified using the CytoHubba plugin.

### 4.8. Subcellular Localization of RhHSF17

The full-length coding sequence (CDS) of *RhHSF17* was amplified and cloned into the pHB binary vector using the restriction enzymes HindIII and PstI. The resulting construct (35S:*RhHSF17*-GFP) and the control vector (pHB-GFP) were introduced into mesophyll protoplasts (leaf-derived) of *A. thaliana* via polyethylene glycol (PEG)-mediated transformation, following the protocol described previously [60]. After 48 h of incubation, GFP fluorescence was observed using a Zeiss LSM 710 confocal laser scanning microscope. The excitation wavelength for GFP was set to 488 nm, and emission was collected between 510 and 530 nm. The assay was repeated three times to ensure reproducibility and the consistency of the results.

### 4.9. Transcription Activation Assay

The full-length coding sequence (CDS) of *RhHSF17* was amplified and inserted into the pGBKT7 vector using restriction cloning. The resulting construct (pGBKT7-*RhHSF17*, referred to as BD-*RhHSF17*), along with the positive control (BD-VP16) and the empty vector (BD), was transformed into *Saccharomyces cerevisiae* strain Y2HGold (TaKaRa, Dalian, China) using the lithium acetate method. Transformed yeast colonies were selected on synthetic dropout (SD) medium lacking tryptophan (SD/-Trp). For the activation assay, serial tenfold dilutions (10^0^, 10^−1^, and 10^−2^) of yeast cultures were spotted onto both SD/-Trp and SD/-Trp-His (SD medium lacking tryptophan and histidine) plates. After incubation at 30 °C for 3 days, growth was assessed and photographed.

### 4.10. Heat Stress and ABA Treatments

Tissue-cultured seedlings of *R. hybrida* ‘Samantha’ were acclimated for experimental treatments. After 60 days of in vitro growth, seedlings were gently removed from culture vessels, and residual medium was rinsed from the roots with sterile water. The seedlings were then transferred to pots containing a 1:1 (*v*/*v*) mixture of peat and perlite. Plants were grown in controlled-environment chambers under a 16 h light/8 h dark photoperiod at 23 ± 1 °C, 70% relative humidity, and a light intensity of 100 μmol m^−2^ s^−1^ provided by cool-white fluorescent lamps.

For heat treatment, 90-day-old ‘Samantha’ seedlings were transferred to a growth chamber set at 42 °C. Leaf samples were collected at 0, 3, 6, 9, and 12 h after the onset of heat stress. For ABA treatment, 90-day-old ‘Samantha’ seedlings were uniformly sprayed with 100 μM abscisic acid (ABA) solution, and leaf tissues were harvested at 0, 3, 6, and 12 h post-application. All samples were immediately frozen in liquid nitrogen and stored at −80 °C until RNA extraction.

### 4.11. Quantitative Real-Time PCR (qRT-PCR) Analysis of RhHSF17

Total RNA was extracted from leaves of *R. hybrida* ‘Samantha’ subjected to heat, drought, and abscisic acid (ABA) treatments using the RNAprep Pure Plant Kit (Tiangen, Beijing, China), following the manufacturer’s instructions. RNA concentration and purity were assessed using a NanoDrop 2000 spectrophotometer (Thermo Scientific, Waltham, MA, USA). For cDNA synthesis, 1 µg of total RNA was reverse-transcribed using the PrimeScript RT Reagent Kit with gDNA Eraser (Takara, Dalian, China). qRT-PCR was performed with TB Green Premix Ex Taq II (Takara, Dalian, China) on an ABI 7500 Real-Time PCR System (Applied Biosystems, Foster City, CA, USA). The thermal cycling conditions were as follows: initial denaturation at 95 °C for 10 min, followed by 40 cycles of 95 °C for 5 s and 60 °C for 40 s. Each reaction was conducted with three biological replicates and three technical replicates. *RhUBI2* was used as the internal reference gene [61]. Gene-specific primers were designed using Primer-BLAST and are listed in Supplementary Table S11. Relative expression levels of *RhHSF17* were calculated using the 2^−ΔΔCt^ method [62].

### 4.12. Generation of Transgenic Arabidopsis Overexpressing RhHSF17

The full-length coding sequence of *RhHSF17* was amplified from *R. hybrida* cDNA and cloned into the pHB vector under the control of the CaMV 35S promoter. The construct was introduced into *A. thaliana* (ecotype Col-0) via the floral dip method using *Agrobacterium tumefaciens* strain GV3101 and subsequently transformed into *Arabidopsis* via the floral dip approach [63]. Transgenic lines were selected on 50 mg∙L^−1^ hygromycin-containing medium and verified by RT-PCR (Supplementary Table S11). Three independent T3 homozygous lines (OE-3, OE-4, and OE-6) with high expression levels were used for further analyses.

### 4.13. Drought Treatment at the Seedling Stage in Arabidopsis Transgenic Lines

For drought tolerance assays at the seedling stage, three-week-old Arabidopsis seedlings grown in soil were subjected to drought stress by withholding water for 12 days, followed by rewatering for 4 days. Phenotypic changes were documented through photography, and seedlings were subsequently harvested for physiological measurements. Fresh weight of aerial parts was recorded immediately after treatment. Additionally, detached leaf water loss rate was measured by placing rosette leaves in a controlled environment, with their weight measured every hour for 7 h to calculate the rate of water loss. The survival rate was recorded based on 20 plants per replicate, while the fresh weight of aerial parts and detached leaf water loss rate were measured using 6 plants per replicate. Data were expressed as mean ± standard deviation (SD). Statistical significance between transgenic and wild-type lines was assessed using Student’s *t*-test, with *p* < 0.05 considered significant and *p* < 0.01 highly significant.

### 4.14. Primer Information

All primer sequences used for cloning, yeast activation assays, qRT-PCR, and transgene verification are provided in Supplementary Table S11.

## 5. Conclusions

This study provides a comprehensive analysis of the HSF gene family in *R. hybrida*, focusing on its roles in heat and drought stress responses, addressing a critical gap in the molecular understanding of stress tolerance in tetraploid cultivated roses. By identifying 71 RhHSF genes and analyzing their phylogeny, conserved motifs, and expression profiles, we provide new insights into the stress-responsive mechanisms in roses. Notably, this work highlights the key role of class C HSFs, such as *RhHSF17*, in integrating stress and hormone signaling pathways, which had not been fully explored in ornamental species. The functional characterization of *RhHSF17*, particularly its induction by both heat and drought stress, offers novel insights into the stress adaptation mechanisms. These findings provide a foundation for the molecular breeding of stress-resilient rose cultivars, with potential applications in ornamental horticulture under changing climate conditions. Additionally, the study underscores the importance of segmental duplication in the expansion of the *RhHSF* gene family, shedding light on its evolutionary dynamics across plant species.

## Data Availability

The transcriptome data from various tissues of *Rosa hybrida* cv. ‘Samantha’ are available under BioProject accession number PRJNA1108167. The RNA-seq data from heat and drought stress treatments are deposited under BioProject accession number PRJNA1090540, and PRJNA1295141. The original contributions presented in this study are included in the article/Supplementary Materials. Further inquiries can be directed to the corresponding author.

## Supplementary Materials

The following supporting information can be downloaded at: https://www.mdpi.com/article/10.3390/plants14203167/s1, Figure S1. Complete phylogenetic tree of *RhHSF* proteins from *Rosa hybrida*; Figure S2. Conserved motif analysis of HSF proteins in *Rosa hybrida*; Figure S3. Distribution of putative cis-acting regulatory elements in the 2 kb upstream promoter regions of RhHSFs; Table S1. Identification of HSF genes in *Rosa hybrida*; Table S2. Plant HSF protein sequences used for phylogenetic tree construction; Table S3. Distribution and Functional Annotation of Cis-Acting Elements in RhHSFs Promoters; Table S4. Intra-genomic Collinearity Analysis of RhHSF Genes in *Rosa hybrida*; Table S5. Collinearity Analysis of Gene Pairs Between Arabidopsis thaliana and Rose hybrida; Table S6. Tissue-Specific Expression of RhHSF Genes in *Rosa hybrida* Based on FPKM Values; Table S7. FPKM-based expression profiles of RhHSF Genes under heat stress at different time points; Table S8. FPKM-based expression profiles of RhHSF Genes under drought stress at different time points; Table S9. STRING Protein ID to Gene Name Mapping for RhHSFs; Table S10. Protein–Protein Interaction Network of RhHSF Genes; Table S11. Primers Used in This Study.
