# Supplementary material for: Genome-Wide Identification, Phylogenetic Classification, and Expression Profiling of the HSF Gene Family in Rosa hybrida Under Heat and Drought Stress
Source: Plants (Basel). 2025 Oct 15;14(20):3167. doi: 10.3390/plants14203167 (PMC12566850; doi:10.3390/plants14203167)
Supplement: Supplementary file 1 [file plants-14-03167-s001.zip › Supplementary Figures.pdf]

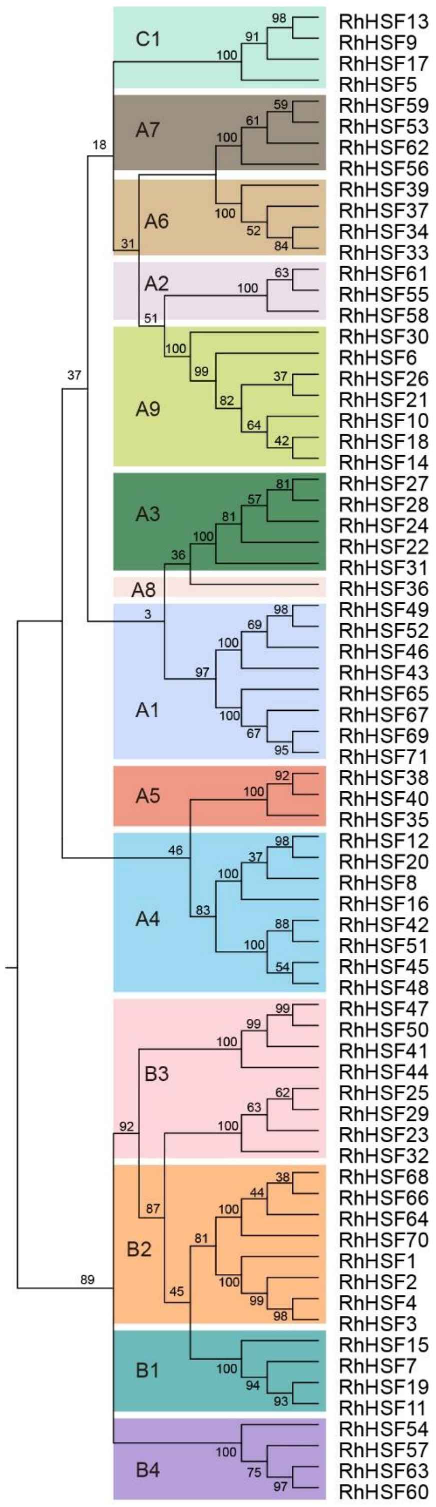

Figure S1. Complete phylogenetic tree of *RhHSF* proteins from *Rosa hybrida*.

The tree includes all bootstrap support values from 1000 replicates, displayed at the corresponding nodes. Subgroup classification (A1–A9, B1–B4, and C1) is highlighted with colored boxes. This figure provides the full bootstrap information corresponding to the simplified tree shown in Figure 2A.

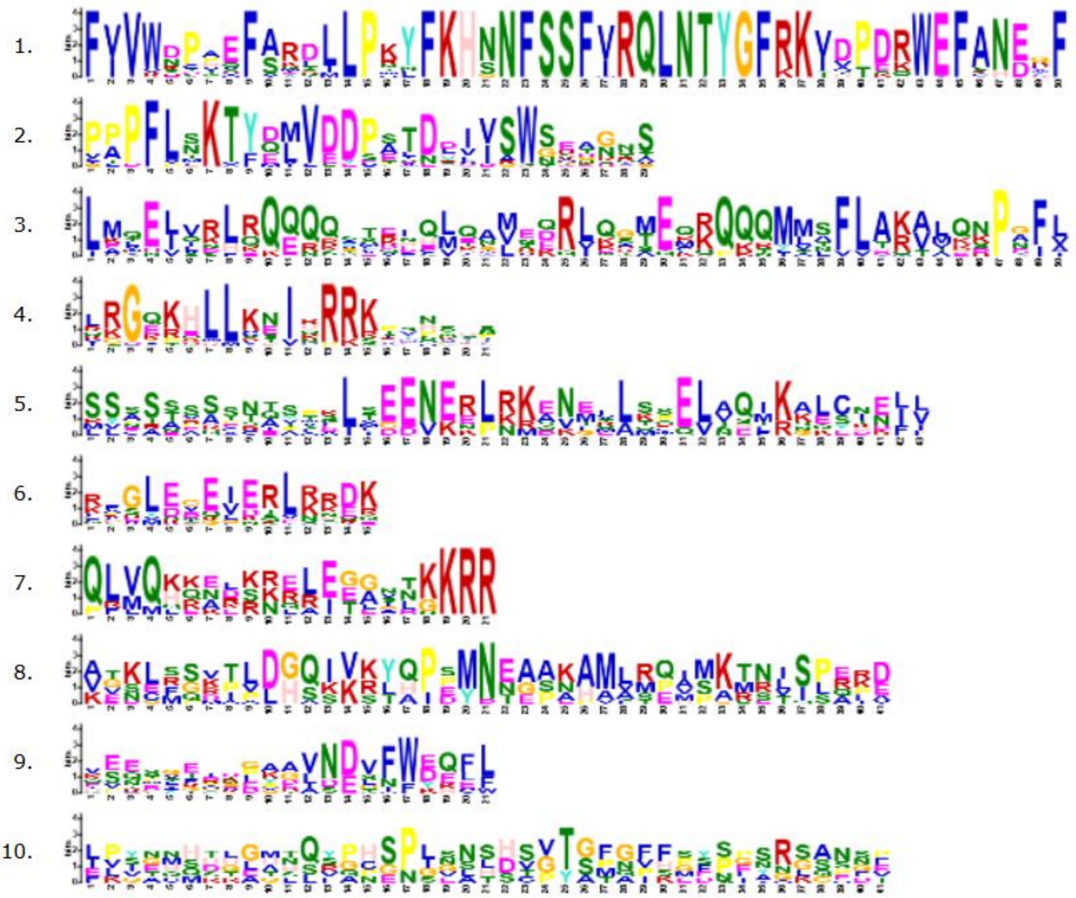

Figure S2. Conserved motif analysis of HSF proteins in *Rosa hybrida*.
Sequence logos of the ten most conserved motifs identified in RhHSF proteins using the MEME suite.

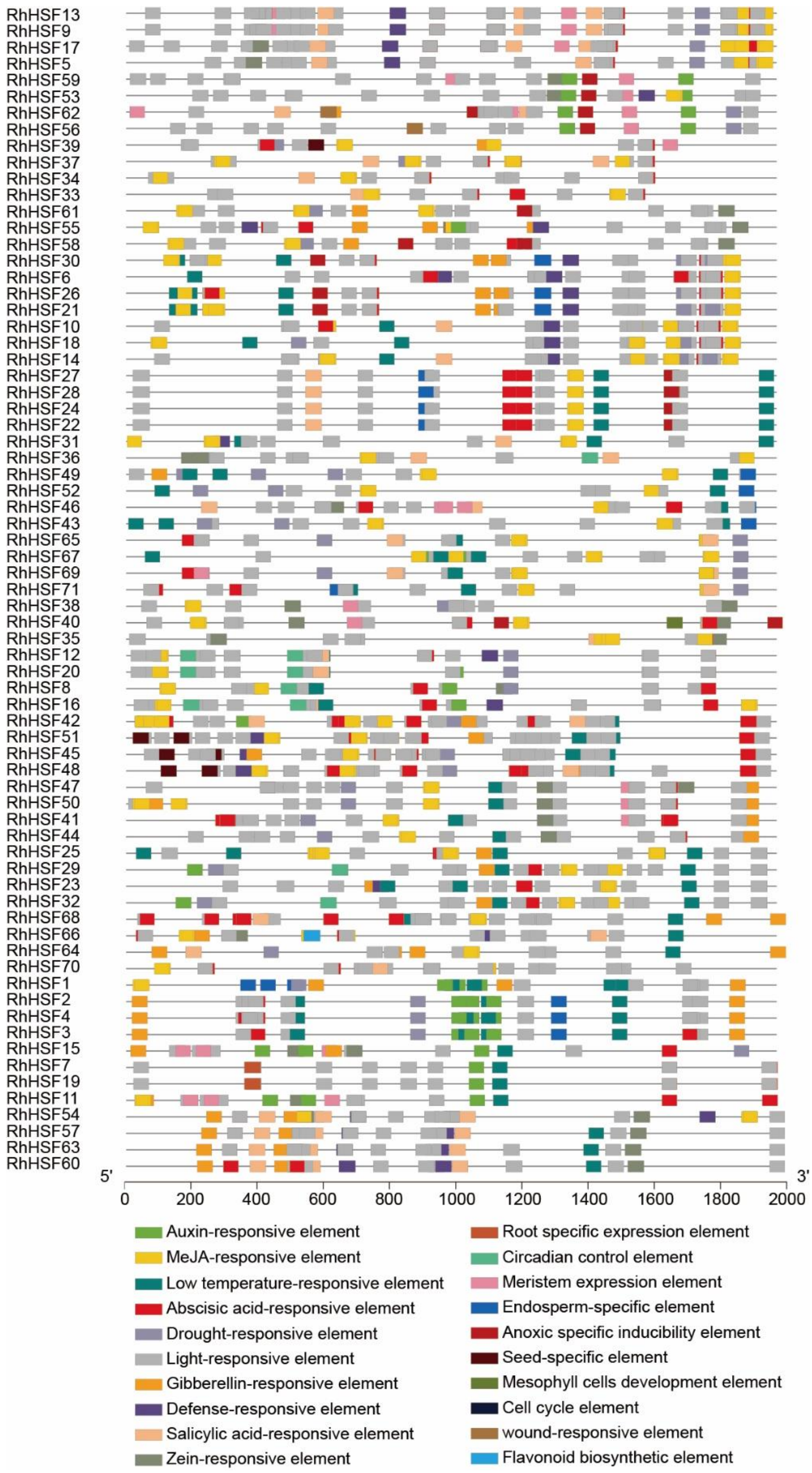

Figure S3. Distribution of putative cis-acting regulatory elements in the 2 kb upstream promoter regions of RhHSFs.

Each colored box represents a specific element type, categorized into four major functional groups: hormone-responsive, stress-responsive, light-responsive, and development-related elements.
